# Nanoclays-containing bio-based packaging materials: properties, applications, safety, and regulatory issues

**DOI:** 10.1007/s40097-023-00525-5

**Published:** 2023-02-02

**Authors:** Kalpani Y. Perera, Maille Hopkins, Amit K. Jaiswal, Swarna Jaiswal

**Affiliations:** 1 School of Food Science and Environmental Health, College of Sciences and Health, Technological University Dublin-City Campus, Central Quad, Grangegorman, Dublin, D07 ADY7 Ireland; 2 Environmental Sustainability and Health Institute, Technological University Dublin-City Campus, Grangegorman, Dublin, D07 H6K8 Ireland

**Keywords:** Nanoclay, Food packaging, Mechanical properties, Barrier properties, Antimicrobial, Biodegradation, Migration, Toxicity

## Abstract

Food packaging is an important concept for consumer satisfaction and the increased shelf life of food products. The introduction of novel food packaging materials has become an emerging trend in recent years, which could be mainly due to environmental pollution caused by plastic packaging and to reduce food waste. Recently, numerous studies have been carried out on nanoclays or nanolayered silicate to be used in packaging material development as reinforcing filler composites. Different types of nanoclays have been used as food packaging materials, while montmorillonite (MMT), halloysite, bentonite (BT), Cloisite, and organically modified nanoclays have become of great interest. The incorporation of nanoclays into the packaging matrix improves the mechanical and barrier properties and at the same time prolongs the biodegradation of the packaging material. The purpose of this article is to examine the development of nanoclay-based food packaging materials. The review article highlights the current state of research on bio-based polymers with nanoclay for food packaging. In addition, the report analyses the mechanical, barrier, and antibacterial characteristics of nanoclay-based food packaging materials. Finally, it discusses the migration of nanoclays, toxicity levels, and the legislation associated with the application of nanoclays.

**Graphical abstract:**

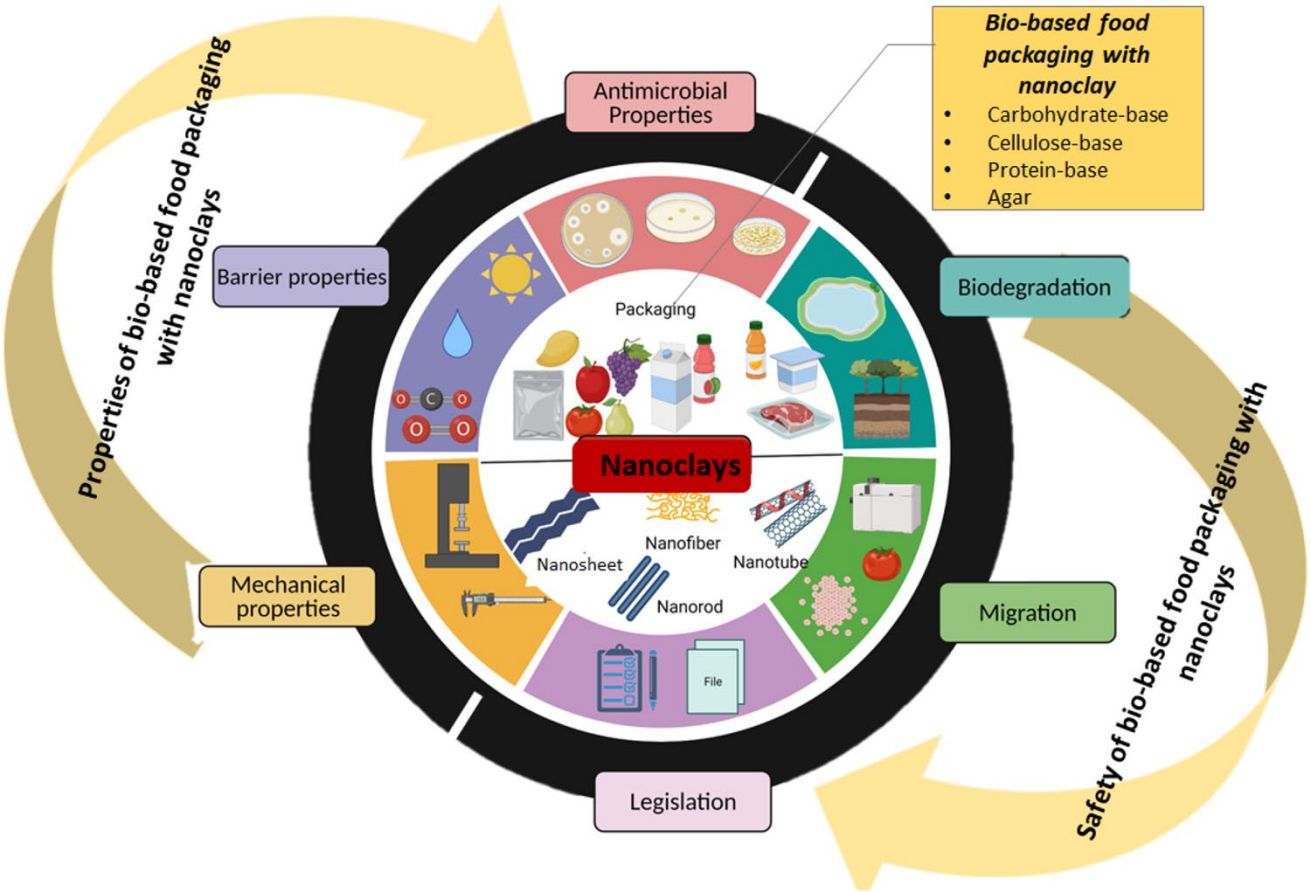

## Introduction

Food packaging serves as an indispensable tool to deliver a food product to the consumer [1]. It increases the product’s shelf life by providing a physical barrier from the adversities in the environment, such as microbial and chemical contaminants, together with facilitating the handling, storage, and transportation of the product [2, 3]. In recent times, a particular focus has been put on the organoleptic characteristics of the product, so it is necessary that packaging can also maintain the colour, flavour, weight, and texture of the food [4].

Traditionally, packaging materials were sourced from plastic, for example, polypropylene is utilized in the packaging of dairy products such as yoghurt containers or sour cream packaging; other commonly used packaging materials include polyamide, polyethylene terephthalate, and ethylene vinyl alcohol. Because of their superior mechanical and barrier qualities, these plastic-based packaging materials are frequently employed in the food packaging sector [1]. However, emerging trends display a shift from the use of these materials to more eco-friendly materials such as bioplastics and biopolymers. This is partially due to the increase in environmental consciousness among consumers and producers, who understand the negative effect of plastic packaging materials on the environment. Furthermore, country-wise legislation such as a ban on single-use plastic exists in countries such as Kenya, Morocco, Senegal, Oman, Albania, Moldova, and Ireland. Further, economic mechanisms, educational strategies, and green procurement have been used to minimize plastic usage [5].

Thus, packaging materials such as bioplastics and biopolymers are currently being explored in the food packaging industry due to their low environmental impact [6]. Such packaging materials that are of current interest are “bionanocomposites” which are composite materials that contain constituent(s) of biological origin and particles with at least one dimension in the range of 1–100 nm. These materials are another developing trend, as they are lightweight, display high-performance activity, and are eco-friendly alternatives to plastic. Nanomaterials are a great choice when designing an alternative to plastic packaging, because they have a larger capacity to enhance the mechanical, thermal, and gas barrier capabilities of biopolymer packaging while keeping their biodegradable characteristics. Nanocomposites have the ability of nanoscale dispersion, which significantly improves the mechanical and physical properties of food packaging [6–8]. Currently, nanomaterials such as zinc oxide, copper oxide, and magnesium oxide are of interest to researchers due to their antimicrobial nature, and at the same time, they have been approved by the European Union (EU) to be used up to a certain limit [9]. For example, specific migration limits are established for other nanoparticles by the EU regulations: Cu = 5 mg/kg food or food simulant, Zn = 5 mg/kg food or food simulant, and Mg = 0.6 mg/kg food or food simulant [6].

The use of nanoclays in food packaging has become of interest due to their importance in improving mechanical properties and barrier properties [10]. Further, nanoclay is abundant, cost-effective, and biocompatible with a diverse morphology and multiple chemical compositions, making it more important over other nanomaterials used in the advancement of materials science and technology. Thus, the applications of nanoclay are not only limited to the food packaging industry, rather it is of great importance in the fields of water treatment, biomedicine, tissue engineering, COVID-19 antibodies, cancer therapy, environmental remediation, energy storage and conversion, electrochemical energy, nanocatalysis, photocatalysis, automotive, nanorobots, and 3D printing [11]. The distinctive platelet form, flaky soft structure, low specific gravity, and high aspect ratio with nanoscale thickness of nanoclays serve as physical indicators of their composition. Their structure allows them to serve as a reinforcement in polymeric packaging materials whilst also reducing the permeability of gases from the product [12].

It was discovered that the mixture of an inorganic nanoclay with an organic polymer matrix creates a polymer nanocomposite. This hybrid differs from regular nanoclay by having a lower mass and also differs from common micro composites, by possessing superior properties [13]. Nanoclay was the first polymer of nanocomposites to come to the market, and it has since become one of the most widely utilized nanomaterials in food packaging [7]. The global nanoclay market size in 2022 was 2.1 billion US dollars and it is predicted to grow up to 6.4 billion US dollars by 2032 [14]. Companies such as Cabot Corp., RTO Company, UBE Industries, Kowa Company, Sun Chemical, Nanocor Inc., Statnano, and Nanoshell LLC use nanoclay in their products [14]. Nanocomposites from nanoclay can be manufactured to form rigid structures such as carbonated drink containers, although they are not limited to these kinds of structures. For example, a commercial nanoclay composite Durethan^®^ KU2-2601 (nanoclay-engineered polyamide film) developed by Bayer was used in beverage packaging [7]. Nanocomposites can also occupy a flexible film form and are used for wrapping fresh and dried food. For example, ‘Debbie Meyer BreadBags™ for bread storage, Aisaika Everfresh Bag for fruits and vegetables, Plantic^®^ Plastic Tray for Cadbury^®^ Dairy Milk™, and Mark & Spencer Swiss Chocolate and beer bottles from Miller Brewing and Hite Brewery Co.’ all use nanoclay nanocomposites in their packaging [13]. Further, Nanocor Inc., USA, produces plastic nanocomposites such as nanoMax^®^-PP-nH, nanoMax^®^-PP, nanoMax^®^-LDPE, nanoMax®-HDPE for food packaging applications. These products had properties such as mechanical resistance, impact resistance, fireproofness and stiffness [15]. Nanoclays also play an important role in active and intelligent packaging; they have been shown to act as antimicrobial agents, be a biodegradability stimulator, and act as a colorimetric indicator template, all of which are being researched for the food packaging industry [12].

**Fig. 1:**
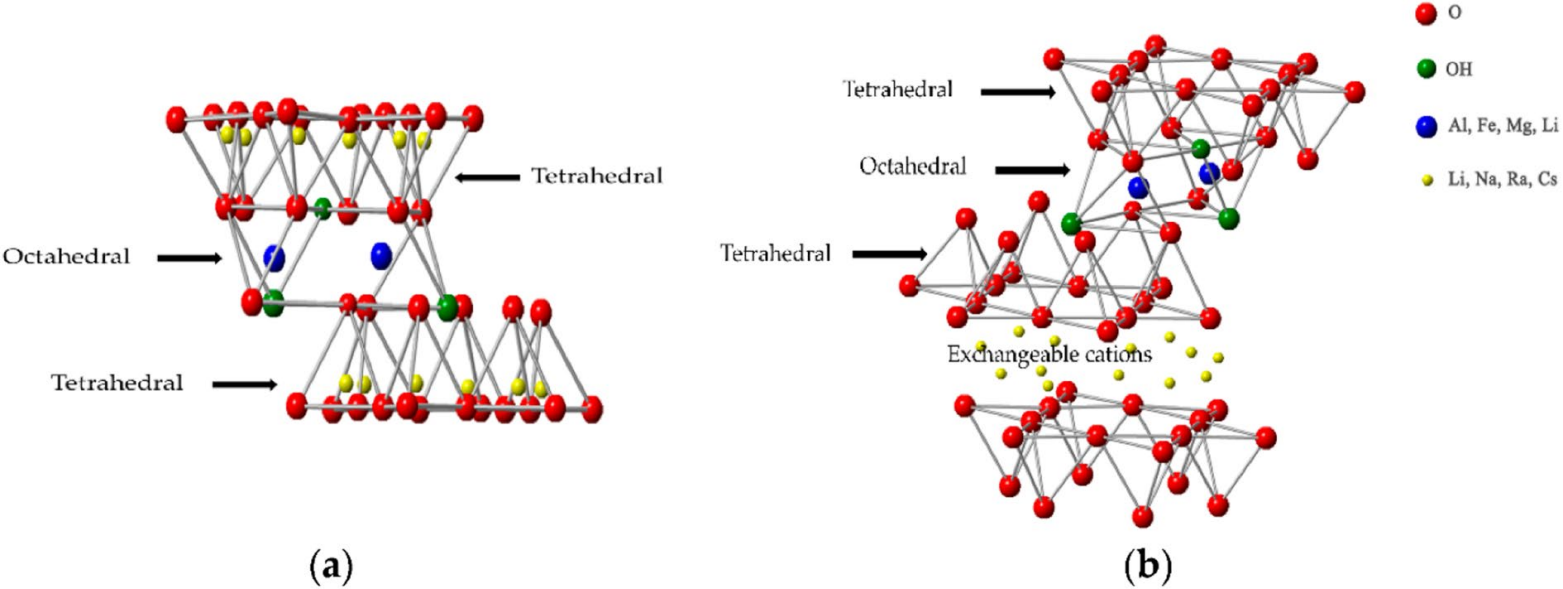
Crystal structures of natural clays. (**a**) Type 1:1 (e.g. kaolinite, halloysite). (**b**) Type 2:1 (e.g. montromorillonite, saponite, BT) [20]

Different nanoclays vary in their characteristics and so are utilized in the packaging of many different products. Natural clays can be broadly classified into two groups based on the layer type, which are (1) 1:1 (the reference plate is formed of the tetrahedral plate and the octahedral plate, e.g. kaolinite, halloysite) and (2) 2:1 (the reference plate is formed of two tetrahedral plates and an octahedral plate, e.g. montromorillonite, saponite, BT) [16]. The crystal structure of these two types of natural clays are shown in Fig. 1. Each tetrahedron in the nanoclay structure comprises a core cation, which is usually silicon (IV) (Si^4+^), synchronized with four oxide anions (O^2−^) that are situated at the vertices. Three basal O^2−^ groups and an apical O^2−^ group are present in each tetrahedron. The tetrahedrons have a propensity to polymerize to form a tetrahedral sheet with varying dimensions, and the three common basal O^2−^ groups can interact with one another. The point of connection with an octahedral sheet is the apical O^2−^. The central metal cation (Mn^+^) of each octahedron is often the aluminium cation (Al^3+^), which is coordinated with six O^2−^ groups at the vertices. An octahedral sheet is created when two adjacent octahedrons share their edges (two O^2−^ or OH groups). Occasionally, lower valence ions with a similar atomic radius will replace the centre Si^4+^ of the tetrahedral sheet and the core Al^3+^ of the octahedral sheet in certain circumstances. Si^4+^ and Al^3+^ are interchangeable for each other as well as for the cations magnesium (Mg^2+^), lithium (Li^+^), and ferrous (Fe^2+^) [17, 18]. MMT, Laponite^®^, and halloysite are amongst some of the most noteworthy and well-researched nanoclays, with MMT and organophilic MMT (OMMT) particularly favoured due to their low cost [19]. Due to their substantial surface area, large aspect ratio (50–1000), and strong compatibility with a variety of organic thermoplastics, MMT and OMMT are utilized for food packaging [12]. This naturally occurring clay consists of many stacked 1 nm nanolayers called tactoids. These tactoids are joined by van der Wall’s interactions, which make it hard to separate them from each other. MMT is often modified to cause swelling of the clay which improves clay dispersion and polymer chain intercalation in the matrix. The delamination of the silicate layers of MMT can be compounded with a polymer to develop a nanocomposite which increases the tensile properties of the substance [20].

Laponite^®^ consists of 1 nm-thick discoid platelets, and the cations in laponite are coordinated by 20 oxide ions and 4 hydroxyl groups with the edges of the disks having –OH groups. Due to its properties, clay platelets can be uniformly dispersed throughout a polymer to create a nanocomposite. Applications of this are seen in laponite containing nanocomposite films for packaging [20]. Sodium hectorite (NaHec), a layered silicate structure, is used to develop food packaging materials with high barrier, biodegradable, and mechanical properties. Due to the biopolymer's organized self-assembly on the nanoclay surface, the nanocomposite has a low free volume and high oxygen and water vapour barrier characteristics, which in turn leads to higher mechanical properties [11]. The addition of polymers affects the phase behaviour of nanoclay solutions, presenting major hurdles in detecting specific interactions between nanoclay and polymer chains. Nanoclay can create physical and chemical interactions with polymer chains through a variety of processes. Electrostatic, hydrogel bonding, ionic bonding, hydrophilic contacts, hydrophobic interactions, and dipole–dipole interactions are some of the potential physical cross-linking interactions between nanoclay and polymer. Electrostatic interactions occur when charged groups on a polymeric backbone make contact with a charged surface/edge of nanoclay. Covalent bonds are detected during chemical interactions between nanoclay and polymers, which arise due to the utilization of active end groups on polymer chains such as dopamine or siloxane. The interactions between nanoclay and polymers are influenced by the polymer structure, molecular weight, type of pendant group, temperature, and hydrophilicity. As polymer molecular weight rises, polymer tails bond amongst multiple nanoclay, forming a physically cross-linked network. The shear-thinning properties of nanoclay are conveyed to the resultant solution if polymer chains are physically adsorbed without the formation of a covalent link. The polymer–nanoclay solution exhibits yielding behaviour only when external stress is applied after bridge stabilization, in which case it is stable regardless of the external ionic concentration [21].

This critical review article is focused on the application of nanoclays in bio-based food packaging material development. Initially, the article highlights the current state of research on bio-based polymers with nanoclay in food packaging. Here, food packaging materials with nanoclay and biopolymers such as carbohydrate, cellulose, protein and agar are discussed. It also discusses the important properties of nanoclays incorporated in food packaging materials such as their mechanical, barrier, and antimicrobial properties. Finally, it discusses the biodegradation of nanoclays, migration of nanoclays, toxicity levels, and the legislation associated with the application of nanoclays.

## Bio-based polymers used in nanoclays-containing composites in food packaging

### Carbohydrate-based polymers

Carbohydrate-based polymers are becoming more prevalent in food packaging due to their affordability, transparency, flavourless and odourless nature as well as low environmental impact. Its drawbacks in comparison to plastic packaging include enhanced water vapor permeability and reduced mechanical properties, which could be due to the hydrophilic nature of polysaccharides [12]. Carbohydrate-based polymers have been preferred to plastic due to their cost competitiveness and reduced environmental pollution. Along with this, it possesses attractive anticoagulant properties and antioxidant activity. In contradiction to these favourable properties, its physicochemical properties lack commercial expectations. For example, it does not possess optimal gas and liquid barrier properties and has poor thermal stability and flexibility; therefore, studies are being conducted to assess the effect of nanoclays on these properties [22].

**Fig. 2:**
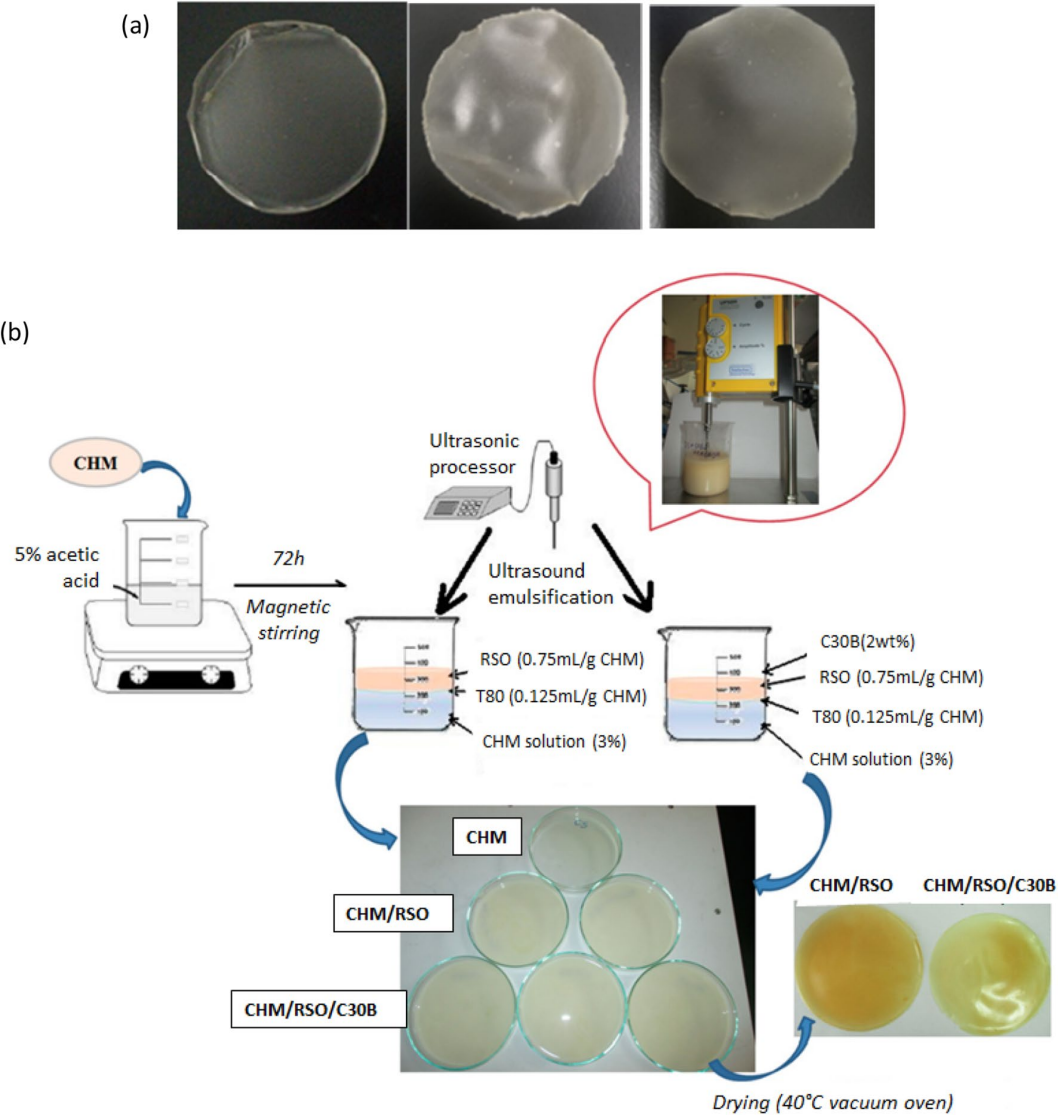
**a** Starch-based packaging films with polyvinyl alcohol and different percentages of MMT nanoclay (0.5, 1.0, and 1.5% w/v, respectively) incorporates [24]. **b** Preparation of chitosan(CHM)-based films with the combination of rosehip seed oil (RSO), MMT C30B, and Tween 80 (T80) by emulsion/solvent casting method [25]

Calambas et al. (2021) designed a starch-based biodegradable packaging film incorporating polyvinyl alcohol and different percentages of MMT nanoclay (0.5, 1.0, and 1.5% w/v) by the solvent casting method with and without ultrasound sonication (Fig. 2a) [23]. The ultrasound-treated films had a more uniform surface when compared with the films without the treatment. The highest barrier properties were observed in the 0.5% w/v nanoclay films with a water vapour permeability (WVP) of 3122.959 g/m^2^.day and a water transmission rate of 331.3667 g/m^2^.day. When considering the mechanical properties, the elongation at break (EB) reduced from 61.39% (0.5% nanoclay) to 34.54% (1.5% nanoclay), while the tensile strength (TS) increased from 3.87 MPa (0.5% nanoclay) to 4.75 MPa (1.5% nanoclay). When analysing the physicochemical properties of the packaging films, 0.5% nanoclay film had the best mechanical and barrier properties when compared to the films with higher nanoclay concentrations. Thus, it has been presented as the best biodegradable film for packaging. The high surface area and nanometric size of the clays enable them to form an efficient interaction for transmitting tensile stresses. A higher concentration of nanoclay film (1.5%) results in the production of aggregates, which degrades the mechanical characteristics.

Further, studies on nanoclay-based packaging materials with starch and chitosan were performed by Jha (2020). The researcher used antifungal agents, potassium sorbate or grapefruit seed extract, to develop the starch–chitosan–MMT packaging film which had antifungal properties. The TS was highest (28.5 MPa) in the starch– chitosan–MMT control film. However, the EB increased significantly with the addition of plasticizers (glycerol/sorbitol), potassium sorbate or grapefruit seed extract. The starch–chitosan–MMT–sorbitol–grapefruit seed extract film exhibited the highest antifungal activity and when bread samples were stored at 25 °C, 59% RH for 20 days [24].

Chitosan is a favourable carbohydrate polymer which is biocompatible and biodegradable. This deacetylated chitin also has excellent film-forming properties and is used in the preservation and packaging of food products. When combined with low-toxic [24] and low-cost nanoclays, this nanocomposite has enhanced functionalities and develops a plastic alternative that is more environmentally friendly. Chitosan and nanoclay combined had a favourable impact on the packaging film’s mechanical, barrier, and antioxidant qualities [26].

Many studies based on chitosan biopolymer have been focused on with the addition of other active agents such as essential oil or nanoparticles to improve the antimicrobial activity of the food packaging films. A chitosan-based food packaging material was also developed by incorporating MMT–copper oxide (MMT–CuO) NPs. MMT alone has significantly improved mechanical, barrier, and optical properties when added to a chitosan matrix. However, it does not have antimicrobial properties. Therefore, chemical modifications of MMT with the combination of CuO nanoparticles enhance the antimicrobial properties. Firstly, the MMT–CuO was prepared by combining MMT and CuSO_4_·5H_2_O by a basic ion-exchange method. The chitosan-based film was prepared based on the casting method with the incorporation of different concentrations of MMT–CuO (1, 3, and 5% w/w) nanocomposites. The addition of a 3% w/w MMT–CuO to the films enhanced the TS and EB values by 58.5 and 52.4%, respectively, while lowering the WVP and oxygen permeability (OP) by 55 and 32%, respectively. This film had strong antibacterial activity against *S. aureus* and *B. cereus* due to the presence of the antimicrobial agent CuO [27]. Further, studies on chitosan-based food packaging materials and nanoclay were performed by Butnaru et al. [25]. As seen in Fig. 2b, biopolymer nanocomposite films were made by casting film-forming emulsions composed of chitosan, Tween 80, rosehip seed oil, and MMT C30B. The biocomposites’ strength at break improved with the addition of C30B nanoclay. The film successfully inhibited *E. coli*, *S. typhymurium*, and *B. cereus* because it contained rosehip seed oil [27].

Edible coatings has also been developed using chitosan and MMT. Here, pears were coated using 1% v/v chitosan and altered concentrations of MMT (0.5%, 1.0%, 2.0% and 4.0% w/w). When compared to the controlled coating (pure chitosan), the nanocomposite coatings had higher thermal stablility, low water solubility, and superior water vapour barrier properties. Through the initial studies it was found that the addition of 0.5% w/w MMT was effective to increase these properties, while maintaining the hydrophilic behavior on the pear surface. The coating was uniform and had no cracks or bubbles. 0.5% w/w MMT was able to increase the shelf life of pear up to 15 days with reduced microbial growth [28].

Dogaru et al. [22] synthesized κ-carrageenan-based bionanocomposite films reinforced with bentonite (BT). In this study, bentonite was used due to its superior sorbent property and swelling capacity. The nanocomposite films were prepared by mixing 3% wt κ-carrageenan with BT powder in different ratios to reach final concentrations of 0% BT, 5% BT, 10% BT, and 15% BT. Once homogenized and set, the film's properties were analysed. When κ-carrageenan and BT were combined, Fourier-transform infrared spectroscopy (FTIR) examination revealed no significant changes in the functional groups of any of the nanocomposite films, indicating that the structure of κ-carrageenan was unaltered. The FTIR also confirmed the interaction between carrageenan and BT via hydrogen bonding and/or electrostatic interactions. Whilst maintaining the κ-carrageenan structure, the BT lowered the film's water uptake due to the hydrogen bonding and thus improved the film’s mechanical properties [22]. Further studies on κ-carrageenan nanocomposite film was performed by Nouri et al. (2020), where MMT (3% w/w) and natural antimicrobial agent *Zataria* multiflora plant extract (1–3% v/v) was incorporated into the matrix. The developed films had better UV barrier properties and higher EB and TS when compared to the κ-carrageenan and MMT films. Further, there was high antimicrobial activity against *E. coli* and *P. aeruginosa*. Thus, the addition of *Zataria* multiflora plant extract can further increase the useful properties of the food packaging film with addition of antimicrobial activity [29].

Sodium alginate is another carbohydrate polymer that has been studied in conjunction with nanoclay for food packaging. MMT nanoparticles (3% w/w) and marjoram essential oil (MO) (0.5, 1.0, and 1.5% v/v) were incorporated into a sodium alginate matrix. The combined effect of MMT and MO on the structural, physical, mechanical, and antimicrobial properties were studied. This film resulted in a 47% decrease in the film's water solubility, although data also exhibited a decrease in the film’s TS upon introduction of MMT. Further, the combination of MMT, MO (1.5% v/v), and SA resulted in the successful inhibition of *E. coli*, *L. monocytogenes*, *B. cereus*, and *S. aureus*. Thus, the combined effect of MMT and MO can be used to form an antimicrobial film with other improved properties [30]. Other food packaging material was developed while using sodium alginate as the matrix incorporating cinnamaldehyde-loaded halloysite nanotubes (T-HNTs) [31]. Films used for food packaging were able to exhibit improved physical functionality and antibacterial activity because of this modified nanoclay.

The TS of the films was observed to be 66.4 MPa, 77.3 MPa, and 80.2 MPa for sodium alginate, sodium alginate–T-HNTs and sodium alginate–cinnamaldehyde-loaded T-HNTs, respectively. T-HNTs were able to increase the TS due to the strain-induced arrangement of nanoclay on the sodium alginate matrix, where the cinnamaldehyde further increases the TS and the EB. While the EB was 2.76%, 2.66% and 2.97% for sodium alginate, sodium alginate–T-HNTs and sodium alginate–cinnamaldehyde-loaded T-HNTs, respectively. The antimicrobial controlled release experiment confirmed the release of cinnamaldehyde from the matrix and the antimicrobial efficiency of the food packaging material.

Other carbohydrate polymers such as *Salvia macrosiphon* seed mucilage matrix and nanoclay (Cloisite 15A) were used to formulate an edible food packaging film [32]. Food-grade edible films were created using the nanocomposite because it contains *Salvia macrosiphon*. The inclusion of nanoclay improves the composite films' mechanical characteristics while reducing their permeability. Since it had an inferior level of water vapour permeability, stronger TS, EB, and reasonable thermal properties compared to the other samples and control film with *Salvia macrosiphon*, the film made at 2% (w/w) of nanoclay was determined to be the best formulation. These films also exhibited antibacterial properties. As a result, it can be utilized as an innovative, antimicrobial edible food packaging film.

As discussed by all the above studies, the barrier properties and mechanical properties of the carbohydrate-based polymers enhanced significantly with the addition of different types of nanoclay. Thus, the main demerits of carbohydrate-based polymers, which have low mechanical and barrier properties, are overcome by the addition of nanoclay. Other active agents such as essential oils or nanoparticles are embedded into this matrix to enhance the antimicrobial or the antifungal properties of the food packaging materials. Carbohydrate polymers-based edible films have also been developed which are highly available, eco-friendly, nontoxic [33] and biodegradable.

### Cellulose-based polymers

Cellulose-based polymers have attracted the attention of researchers over the past few years due to their low density, non-abrasiveness, combustibility, non-toxicity, biodegradability, and low cost. However, unappealing characteristics such as poor interfacial interaction and significant water retention reduce the value of cellulose fibre packaging alone [34].

Zheng et al. (2019) assessed the oxygen and water barrier properties of cellulose nanofibril (CNF) films for food packaging containing two kinds of BT: PGN (platelet aspect ratio 300–500) and PGV (platelet aspect ratio 150–200) at different concentrations (15, 30, and 45 wt %). When incorporated into the continuous CNF matrix, both types of BT lowered the film’s degradation temperature and TS (in the most extreme case, it was lowered by 30 MPa when 45% PGN was present); it is hypothesized that these results were due to a partial disruption of the CNF H-bond network. A contact angle analysis was performed by polar and nonpolar liquids, and its results suggested that the PGN-containing films were more hydrophilic. It was also found that the presence of PGN reduced the 15 wt % water vapour transmission rate (WVTR) from 425 to 375 g/m, although the higher proportions of BT had negative effects on this trend. PGN also induced improvements in the film’s oxygen transmission rate (OTR), as it restricted the oxygen passage in the dry state and at higher relative humidity (although to a lower extent). For example, 15% PGN reduced the film’s OTR by 47% at a relative humidity of 50%. Overall, this study suggests that the introduction of BT into CNF films has positive effects on its water/oxygen penetration despite a partial reduction in the film’s mechanical properties [34].

Mirmehdi et al. [35] evaluated the effect of a spray on CNF and nanoclay hybrid layer on the properties of food packaging paper to create a multilayer composite. It was found that introducing nanoclay to the CNF medium improved the film's gas barrier property to oxygen and water vapour. The increased gas barrier capability was attributed to the highly ordered nanoplatelet-like structure of the nanoclay preserved in the composite with CNF. This structure makes the diffusion of gas molecules difficult and hinders permeability. The study also found that an increased coating weight decreased the WVTR. The uncoated control paper had a WVTR of 28.5 g/m^2^ /24h^1^, whilst the coated paper had a WVTR of 4 g/ m^2^ /24 h^1^ at 25 °C with 50% relative humidity; this equates to an 86% reduction in WVTR with 29 μm of added nanocomposite layer. When considering the mechanical properties, the TS of the uncoated control paper was ∼ 6.3 KN/m. The TS rose in all coated paper samples (30 S or 60 S) when compared to the control untreated samples. However, with the increasing concentration of nanoclay (0–5%,), the TS decreased from ∼ 7.5 KN/m to ∼ 6.5 KN/m for 30 S and from ∼ 7.7 KN/m to ∼ 6.7 KN/m for 60 S coated paper sample. Thus, the coating improves both the barrier properties and the mechanical properties, whereas, increased nanoclay concentration reduces the TS of the paper.

When considering the cellulose-based food packaging material, it can be concluded that nanoclay influences the properties of the packaging material, especially by improving the barrier and the mechanical properties. Thus, the demerits of high water absorption can be reduced through increase in the barrier properties as a result of nanoclay addition [35].

### Protein biopolymers

Protein biopolymers are used for the formation of packaging film, and the properties of each film vary depending on where the protein is sourced from. Protein biopolymers can be classified into animal proteins such as casein, whey, and gelatine and plant proteins including gluten, soy proteins, and zein. These protein biopolymers provide excellent gas barrier qualities as well as adequate mechanical properties in packaging films. Protein-based films, on the other hand, have poor water barrier properties due to their hydrophilicity. Protein films alone are unable to withhold these characteristics in comparison to plastic packaging and so nanoclay composites are introduced [36].

Nanocomposites are considered a favourable alternative to plastic, as they have an ability for nanoscale dispersion; this brings significant improvement in the mechanical and physical properties [37]. In this study, gelatine, nanofilms containing *Agave angustifolia* Haw microfibres (MF) were produced using a thermo compression technique. Primarily, the surface and cross-sectional morphology of the films were assessed, and it was noted that as the MF and BT concentration in the packaging increased, roughness, pores, and homogeneities were also increased. The permeability of water molecules was positively affected by the increased tortuosity inside the films caused by the presence of MF and BT. Additionally, while increasing the film's TS and elasticity, the inclusion of the nanoclay decreased the EB. For instance, there was a 64% increase in TS between the control M1 (0 g/100 g BT) and sample M10 (5.5 g/100 g), whilst the EB reduced 58% from M1 to the lowest value at M10. The inorganic nature of BT also improved the particle's thermal stability. FTIR study showed proper compatibility and synergy between the MF and BN particles with the gelatine matrix. This study further confirms the possibilities of nanoclay protein-based polymers for food packaging [37]. Also, as in the studies of Shams et al. (2019), whey protein isolate–gelatine nanocomposites–natural orange peel extract and Cloisite 30B film improved the water barrier properties and mechanical properties with the inclusion of nanoclay [38]. As a result, nanoclay helps to enhance the crucial characteristics required for food packaging, paving the way for the use of protein-based biopolymers in the packaging sector in the future. Protein biopolymers usually have good mechanical properties, which are further increased with the addition of nanoclay. Most importantly, the low water barrier properties are enhanced with the addition of nanoclay.

### Agar

Agar is a carbohydrate manufactured by the *Rhodophyceae* red algae, which are composed mainly of agarose and agaropectin. Agarose is a neutral, linear molecule, whilst agaropectin is a non-gelling unit that is charged, sulphated, and branched. Although more and more agar-based food packaging materials are emerging, their applications in the food industry are limited due to their poor mechanical properties and low thermal stability. These limitations interfere with the food packaging film manufacture throughout processing and handling. They also do not aid in the extension of the shelf life of the product and the food quality, as they have poor antibacterial activity and optical properties. These properties can be improved through combining agar with nanoclay. The nanofillers interact closely with the component due to their high surface-to-volume ratio, which improves the mechanical, thermal, and barrier qualities of the packaging film [38].

Palem et al. [39] prepared a nanocomposite film consisting of carboxymethylcellulose, polyvinylpyrrolidone, agar, and nano sepiolite clay (using varying concentrations of 0, 0.3, 0.5, 0.7, 0.9, and 1.5%) to test the effects the clay would have on the agar-based film. The films were characterized by structural, morphological, and thermal properties. Upon analysis of the results, it was found that the films containing 1.5% nano sepiolite had a higher TS of 78.8 MPa than the film without nanoclay (53.2 MPa). Further, the films containing nanoclay also displayed improved thermal properties [38, 39].

In another study, a combination of agar, gellan gum, and MMT (0, 2.5, 5.0, 7.5, and 10 wt %) was used as food packing film using a solution casting method. The film was later assessed for the effects of the MMT on the microstructural, rheological, mechanical, thermal, ultraviolet, and water barrier properties of the film. The result found that the TS increased by 14.1 MPa in the presence of MMT nanoclay (10%wt), whereas the film’s water barrier was reduced from 1.9 ± 0.18 (× 10^−9^ g/m^2^ Pas) to 1.70 ± 0.11 (× 10^−9^ g/m^2^ Pas) and the contact angle reduced from 56.8° to 49.4° upon the incorporation of the 10wt% MMT in comparison with 0%wt MMT. This nanocomposite also displayed viscosity reduction at a high rate along with shear-thinning behaviour [38]. Aligning with the results of the other biopolymers in nanoclay-based food packaging materials, nanoclay also improves the properties of agar. Through the studies, it was discovered that the use of nanoclay increased the mechanical, barrier, and thermal properties.

The recent applications of nanoclay composites in food packaging are highlighted in Table 1.

**Table 1:** Recent applications of nanoclay composites in food packaging

Serial no.	Type of nanoclay	Packaging materials	Packaged food product	Characteristics of the packaging material	Benefits compared to conventional packaging	References
1	BT	Poly(lactic acid)	N/A	Active food packaging material	Active properties, mechanical properties, barrier properties	[40]
2	BT	Xylan–alginate	N/A	Edible films	Biodegradable, edible, eco-friendly	[41]
3	BT 32A	Carnauba wax	Oranges	Coating to preserve sensory and nutritional quality	Biodegradable, edible, eco-friendly, enhanced shelf life	[42]
4	Cloisite 20A	Starch–low-density polyethylene–date palm seed extract	N/A	Antimicrobial active packaging film	Biodegradable, eco-friendly, antimicrobial	[43]
5	Cloisite Na^+^	Polyvinyl alcohol	N/A	Base paper coating	Mechanical properties, barrier properties	[44]
6	Cloisite 30B	Poly(butylene succinate-co-butylene adipate)–poly(lactic acid)	N/A	Multinanolayer nanocomposites	Biodegradable, eco-friendly, mechanical properties, barrier properties	[45]
7	Cloisite 20A	Polypropylene -based cellulose nanofiber	N/A	Active packaging material	Mechanical properties, barrier properties, active properties	[46]
8	Cloisite 30B	Gelatin–whey protein isolate–orange peel extract	N/A	Antimicrobial food packaging material	Biodegradable, eco-friendly, antimicrobial, mechanical properties, barrier properties	[47]
9	Halloysite	Xylan–alginate	N/A	Edible films	Biodegradable, eco-friendly, edible	[41]
10	MMT	Chitosan	Gouda cheese	Antimicrobial active food packaging	Biodegradable, eco-friendly, antimicrobial, mechanical properties, barrier properties	[48]
11	MMT	Rice flour–gelatin	Pork belly	Antimicrobial active food packaging extended shelf life	Biodegradable, eco-friendly, antimicrobial, mechanical properties, barrier properties	[49]
12	MMT	Low-density polyethylene	Sugarcane juice	Active packaging film to extended shelf life	Antimicrobial, mechanical properties, barrier properties	[50]
13	MMT	Chitosan–CuO	N/A	Antimicrobial	Biodegradable, eco-friendly, antimicrobial, mechanical properties, barrier properties	[26]
14	MMT	Pectin	N/A	Bilayer food packaging material	Biodegradable, eco-friendly, mechanical properties, barrier properties	[51]
15	MMT	Wheat gluten	N/A	Active food packaging material	Biodegradable, eco-friendly, antimicrobial, mechanical properties, barrier properties	[52]
16	MMT	Poly(butylene succinate-co-butylene adipate)–poly(lactic acid)–carvacrol	N/A	Antimicrobial active packaging film	Antimicrobial, mechanical properties, barrier properties	[53]
17	MMT Cloisite C30B	Chitosan–rosehip seed oil	N/A	Antimicrobial active packaging film	Biodegradable, eco-friendly, antimicrobial, mechanical properties, barrier properties	[25]
18	MMT	Gelatin–chitosan Chitosan	N/A	Active food packaging material	Biodegradable, eco-friendly, antimicrobial, mechanical properties, barrier properties	[54]
19	MMT	Poly(lactic acid)–thymol	N/A	Antimicrobial and antioxidant active packaging film	Biodegradable, eco-friendly, active properties, mechanical properties, barrier properties	[55]
20	MMT-Na^+^	Polyvinyl alcohol	N/A	Packaging Coating against moisture blocking	Barrier properties	[56]
21	Nanoclay	Pectin–methylene blue	Oranges, tangerines and kiwi	Antioxidant and smart active film to measure vitamin C levels	Biodegradable, eco-friendly, antioxidant, mechanical properties, barrier properties, intelligent properties	[57]
22	Nanoclay	Starch–tragacanth gum	N/A	Active food packaging material	Biodegradable, eco-friendly, antimicrobial, mechanical properties, barrier properties	[58]
23	Nanoclay	Poly(lactic acid)	N/A	Active food packaging material	Biodegradable, eco-friendly, antimicrobial, mechanical properties, barrier properties	[59]
24	Nanoclay	Starch loaded with methyl orange and bromocresol green	Milk	Intelligent packaging for milk spoilage	Biodegradable, eco-friendly, mechanical properties, barrier properties, intelligent properties	[58]
25	Nanoclay–hydrophilic bentonite	Polyvinyl alcohol–red cabbage extract	N/A	pH-responsive intelligent packaging film	Biodegradable, eco-friendly, mechanical properties, barrier properties, intelligent properties	[60]
26	Nanoclay Cloisite® Na +	Pectin–*Carum copticum* essential oils–β-carotene	Butter	Smart active film to determine oxidation of butter	Biodegradable, eco-friendly, mechanical properties, barrier properties, intelligent properties	[61]
27	Organically modified MMT	k-carrageenan–cellulose nanocrystals	N/A	Active packaging film	Biodegradable, eco-friendly, active properties, mechanical properties, barrier properties	[62]
28	MMT	Cassava starch–clove essential oil	strawberries	Active packaging film	Biodegradable, eco-friendly, active properties, mechanical properties, barrier properties	[63]

*BT*  bentonite, *MMT* montmorillonite

## Properties of nanoclays-containing bio-based packaging materials

### 3.1 Mechanical properties of nanoclays-containing bio-based packaging materials

The mechanical properties of packaging material are essential to improve the food quality and shelf life during handling, storage, and transportation. Bio-based food packaging systems can be designed to have particular mechanical properties depending on their intended function and the product. The primary purpose of incorporating a nanoparticle (reinforcement agent) into a biopolymer matrix is to improve the mechanical properties of the packaging material. The mechanical properties of nanocomposite films are evaluated based on their TS, Young’s modulus (YM), and percent EB. Films rely on the interfacial interaction amid the polymer matrix and nano-sized fillers and need well-dispersed clay layers throughout the polymer matrix. A large aspect ratio of the nanoclay also favours an enhancement in the mechanical properties [16, 64].

Yeasmin et al. [65] studied a solution casting method to produce a cellulose nanofibril-MMT-pullulan nanohybrid film. Different concentration of 0, 0.5, 1, 3, and 5% MMT was utilized for film preparation. The inclusion of MMT enhanced the film's TS, thermal stability, and water barrier qualities while decreasing its susceptibility to moisture. The highest TS was achieved at 5 wt% MMT content with a 45.9 MPa, this is approximately an increase of 10 MPa when compared to the 0 wt% MMT film. A lower moisture uptake was noted with a ~ 3% reduction between the 0% MMT film and the 5% MMT film. Most importantly, these films were highly biodegradable and degraded within 18 days [65].

**Fig. 3:**
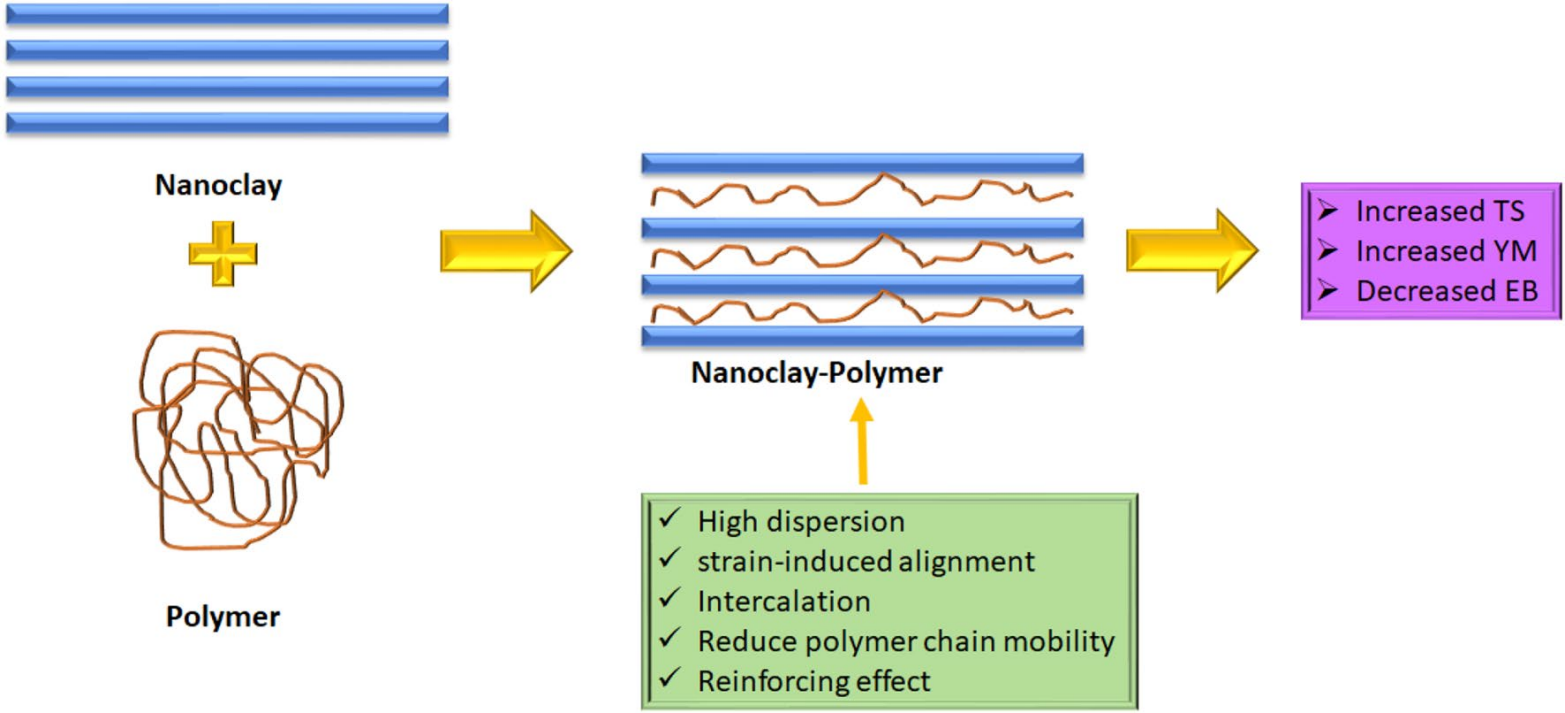
Schematic representation of the influence of nanoclay on the mechanical properties of the polymer matrix. *TS* tensile strength, *YM* Young’s modulus, *EB* elongation at break

The TS of the food packaging materials is increased since nanoclay functions as an efficient reinforcement agent to biopolymers due to its in height dispersion in the biopolymer matrix and the strain-induced alignment of the clay particles with the polymer chains. Additionally, the intercalation of the clay and polymers supports it (Fig. 3) [41]. In addition, the incorporation of nanoclay into bio-based food packaging materials decreases the EB value of the films, and thus overall elasticity of the nanocomposite is reduced by the inclusion of the nanomaterial. This is because nanoclay's stiffening and reinforcing properties create a network of polymeric phases and reduce the mobility of polymer chains as observed by the studies of Radfar et al. [43] highlighted in Table 2 [43]. In addition, the YM decreased; since the volume fraction of nanoclay exceeded the threshold limit value, the fully exfoliated structures were transformed into partially exfoliated–intercalated structures. According to Messin et al. [45], a substantial increase in YM, i.e. approximately 50% for 5 wt% of C30B, is often connected with a good exfoliation and dispersion of fillers inside the polymeric matrix [45]. Further, increase of the YM, i.e. the stiffness of the film can also be due to the reinforcing effect of the packaging matrix [43]. Hence, there is a strong potential for improvements in the mechanical properties of various packaging materials with the introduction of nanoclays, although the concentrations at which the nanoclay is most beneficial are yet to be determined and are thought to vary upon which nanoclay is utilized [16]. In the study by Davachi and Shekarabi [32], with the addition of 2 wt% nanoclay, the TS, YM, and EB all increased; however, with the addition of 3 wt% nanoclay, all mechanical parameters decreased, with the exception of the YM. [32]. The decrease in the TS of the packaging material was observed when the nanoclay concentration was increased because of the agglomeration of nanoparticles in the film matrix [43]. Many other studies also observed that the addition of nanoclays in the packaging materials increases the mechanical properties of the packaging matrix as depicted in Table 2.

**Table 2:** Effect of the nanoclay on the mechanical properties of the bio-based packaging materials

Serial no.	Type of nanoclay	Packaging materials	Concentration of nanoclay (%)	Tensile strength (MPa)	Elongation at break (%)	Young’s modulus (MPa)	References
1	MMT	Sodium caseinate–starch blend	0%w/w	7.72 ± 1.68	30.49 ± 14.05	N/A	[66]
			10%w/w	7.66 ± 1.15	70.81 ± 11.52	N/A	
2	Nanoclay	Flour–gelatine– catechin–lysozyme	0%	3.64 ± 0.77	84.17 ± 9.33	N/A	[49]
			5% w/w	3.88 ± 0.48	95.59 ± 12.55	N/A	
			5% w/w _ catechin–lysozyme	4.56 ± 0.64	97.35 ± 8.70a	N/A	
3	Cloisite 15A	*Salvia macrosiphon* seed mucilage	0	16.35	5.39	327.11	[67]
			0.5%	18.62	5.42	341	
			1%	21.43	5.92	356	
			1.5%	23.38	6.43	374	
			2%	25.84	7.64	392	
			3%	24.15	5.26	402	
4	MMT	Pullulan and tempo cellulose nanofibrils	0, 0.5, 1, 3, 5%	45.9 MPa for 5 wt. % MMT content	N/A	N/A	[65]
5	Cloisite 20A	LDPE–starch– date palm seed extract	0%	3 ± 0.2	10 ± 0.25	100.79 ± 0.11	[43]
			2.5%	9.93 ± 0.03	84 ± 0.15	342.4 ± 0.01	
			5%	9.86 ± 0.05	52.5 ± 0.01	342 ± 0.02	
6	Cloisite 20A	Polypropylene -based cellulose nanofiber	0 wt %	36.44 ± 0.18	416.56 ± 82.83	66.68 ± 0.69	[68]
			1 wt %	34.73 ± 0.56	605.95 ± 13.84	65.65 ± 0.90	
			3 wt %	36.61 ± 0.55	289.79 ± 65.85	67.44 ± 0.55	
			5 wt %	34.62 ± 0.63	499.06 ± 66.34	65.53 ± 0.39	
7	Cloisite 30B	Poly(butylene succinate-co-butylene adipate)–poly(lactic acid)	0 wt%	241 ± 19	1360 ± 148	35 ± 3	[45]
			2 wt%	276 ± 33	1405 ± 67	31 ± 2	
			5 wt%	362 ± 19	1242 ± 97	27 ± 2	
8	BT	Xylan–alginate	0 wt%	8.87 ± 2.05	51.29 ± 5.89	31.91 ± _ 7.04	[41]
			1 wt%	11.52 ± 2.40	40.23 ± 9.61	39.86 ± 5.63	
			3 wt%	11.95 ± 4.12	42.03 ± 14.23	42.91 ± 7.08	
			5 wt%	18.86 ± 3.19	46.70 ± 7.55	56.24 ± 8.98	
9	Halloysite	Xylan–alginate	0 wt%	8.87 ± 2.05	51.29 ± 5.89	31.91 ± 7.04	[41]
			1 wt%	11.11 ± 1.89	46.36 ± 5.10	35.38 ± 5.81	
			3 wt%	8.93 ± 1.35 33	46.99 ± 3.74	28.01 ± 5.00	
			5 wt%	7.20 ± 1.39	44.70 ± 4.06	22.55 ± 5.01	
10	Cloisite 30B	Gelatine–whey protein isolate	0%w/w	5.62 ± 0.01	38 ± 0.01	N/A	[47]
			5%w/w	9.24 ± 0.34	27 ± 1.50	N/A	
11	Nanoclay	Poly(lactic acid)	0 wt%	9.34	8.0	2.8	[59]
			1 wt%	35.3	7.11	8.1	
			2 wt%	36.8	6.85	8.5	
			4 wt%	38.6	5.49	8.7	
			6 wt%	39.9	5.41	9.2	
12	MMT	Pectin	0 wt%	22.3 ± 2.5	4.3 ± 0.3	1027 ± 127	[51]
			2.5 wt%	24.2 ± 4.2	3.7 ± 0.7	1227 ± 215	

*BT*  bentonite, *MMT* montmorillonite

### Barrier properties of nanoclays-containing bio-based packaging materials

Polymer materials with good barrier properties are used in food packaging to avoid the passage of small molecules of gases, moisture, or flavours through the matrix [25]. The WVP or WVTR of the packaging material determines its moisture barrier characteristic, which impacts the shelf life and quality of packed food. One of the biggest flaws in packaging applications is the low moisture barrier characteristic of biopolymer-based materials [67]. The WVP of a packaging film should be low as possible to avoid the transfer of water vapour from the exterior of the food packaging material to the interior [42]. Oxygen permeability (OP) is yet another crucial characteristic of packing materials, which controls how much oxygen is transported across the film to extend the shelf life and improve the quality of the packaged food. The presence of oxygen influences the progression of many reactions such as oxidation involving components responsible for the food's colour and aroma. The materials with the least amount of OP are preferable when packaging oxygen-sensitive items. When compared to plastic packaging, biopolymer-based packaging materials have inferior oxygen barrier qualities. As a result, specific biopolymer changes are required to reduce OP and improve its application in the food packaging application [67]. Several factors of a polymer material influence the OP of the film such as the polymer's internal structure, crystallinity, molecular weight, and molecular entanglement [46]. Oxygen penetration through a material is the calculation of the mass or volume of oxygen that can pass through a known region of a material within a given time period. CO_2_ is a critical barrier feature in food packaging applications since it can initiate degradation events and must be eliminated to preserve the freshness of food goods. Thus, the low CO_2_ permeability of the packaging material is also important.

**Fig. 4:**
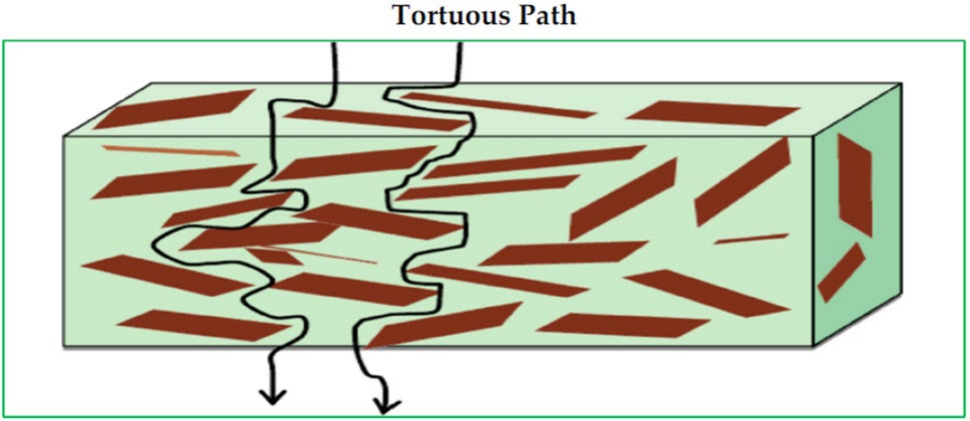
Mechanism of barrier properties improvement from nanoclay incorporation into a matrix by making a tortuous path in the matrix [16]

Consequently, with the aim of improving the barrier properties treatments has been carried out, one of which is the incorporation of nanoparticle into the polymer matrix. Nanoclays have the ability to drastically increase the barrier properties of the polymer matrix (Table 3). As shown in Fig. 4 nanoclays improve polymer barrier properties by creating a maze or a complicated path through the polymer matrix that delays gas molecule migration. The barrier properties are influenced by the aspect ratio of nanoclays distributed in the polymer matrix [16]. The influence of nanoclay on the barrier properties of the food packaging material makes nanoclay a crucial nanoparticle that could be used in food packaging material.

**Table 3:** Effect of the nanoclay on the barrier properties of the bio-based packaging materials

Serial no.	Type of nanoclay	Packaging materials	Concentration of nanoclay	Water permeability	Gas permeability	References
1	Nanoclay	Flour–gelatine– catechin–lysozyme	0%	WVP (× 10^−5^gmm^−1^ h^−1^ cm^−2^ Pa^−1^) 7.85 ± 0.98	N/A	[49]
			5% w/w	10.29 ± 0.08	N/A	
			5% w/w (catechin–lysozyme)	9.81 ± 0.73	N/A	
2	Cloisite 20A	Polypropylene -based cellulose nanofiber	0 wt %	WVP (g mm/m^2^-day-atm) 0.37 ± 0.01	OP (cc-mm/m^2^-Day-atm) 107.02 ± 6.78	[68]
			1 wt %	0.39 ± 0.05	106.71 ± 2.94	
			3 wt %	0.39 ± 0.03	95.77 ± 0.97	
			5 wt %	0.37 ± 0.02	92.95 ± 1.58	
3	MMT Cloisite 30B	Chitosan–rosehip seed oil	0 wt%	N/A	OP (mL/m^2^ per day) 212 ± 10.6 CO_2_ Permeability (mL/m2 per day) 37 ± 1.1	[24]
			3 wt%	N/A	134 ± 6.7 CO_2_ Permeability (mL/m^2^ per day) 18 ± 0.9	
4	MMT	White fleshed pitaya peel pectin–white fleshed pitaya peel betacyanins	0 wt%	WVP (10^–9^ g m^−1^ min^−1^ Pa^−1^) 2.17 ± 0.08	N/A	[69]
			5 wt%	1.92 ± 0.10	N/A	
5	MMT-Na^+^	Polyvinyl alcohol	0 wt.%	Moisture permeability (g cm/(m^2^ day)) 2.05 × 10^–1^	N/A	[56]
			2 wt.%	1.1 × 10^–1^	N/A	
			4 wt.%	6 × 10^–2^	N/A	
			6 wt.%	4 × 10^–2^	N/A	
			8 wt.%	3.2 × 10^–2^	N/A	
			10 wt.%	2.8 × 10^–2^	N/A	
6	BT	Xylan–alginate	0 wt%	WVP (g s^−1^ m^−1^ Pa^−1^) 3.94 × 10^–10^	N/A	[41]
			1 wt%	2.48 × 10^–10^	N/A	
			3 wt%	2.33 × 10^–10^	N/A	
			5 wt%	2.01 × 10^–10^	N/A	
7	Halloysite	Xylan–alginate	0 wt%	WVP (g s^−1^ m^−1^ Pa^−1^) 3.94 × 10^–10^	N/A	[41]
			1 wt%	2.68 × 10^–10^	N/A	
			3 wt%	2.34 × 10^–10^	N/A	
			5 wt%	2.01 × 10^–10^	N/A	
8	Nanoclay	Poly(lactic acid)	0 wt%	WVTR (g mm/m^2^ day) 181.8	OP Coefficients (cm^3^ mm/m^2^ day bar) 488.9	[59]
			1 wt%	97.2	239.4	
			2 wt%	87.7	209.3	
			4 wt%	72.3	183.6	
			6 wt%	58.1	186.1	
9	MMT	Pectin	0 wt%	WVP (10^–11^ mol/m s Pa) 2.15	OP (10^–16^ mol m/m^2^ s Pa) 1.12	[51]
			2.5 wt%	1.45	0.82	

*BT* bentonite, *WVP* water vapour permeability; *N/A* not applicable; *OP* oxygen permeability; *MMT* montmorillonite; *WVTR*  water vapour transmission rate

Motamedi et al. [42] designed a packaging film containing rice flour–gelatine with nanoclay. In this study, the nanoclay containing film (9.57–10.29 × 10^− 5^ g mm/ h/ cm^2^ /Pa) showed higher WVP when compared to the control films (7.85 × 10^− 5^ g mm/ h/ cm^2^ /Pa). Thus this packaging is not suitable for dry food product applications [42].

Further studies on nanoclay's influence on the barrier properties were conducted by Jung et al. (2020). A food packaging material was developed from polypropylene, cellulose nanofiber, and nanoclay (Cloisite 20A). The barrier properties of the film were analysed by measuring OP by an OTR analyser and testing WVP by a WVTR analyser. The OP had reduced from 132.66 ± 0.79 (control) to 92.95 ± 1.58 cc-mm/m^2^-day-atm with the addition of 5 wt% nanoclay. When the nanoclay concentration is increased the OP increases as a result of the formation of a more tortuous path inside the polymer. While the WVP was reduced slightly from 0.38 ± 0.02 (control) to 0.37 ± 0.02 g.mm/m^2^.day.atm with the addition of 5 wt% nanoclay. Both the polymer and the inclusion of nanoclay showed excellent WVP [68].

Results of a different study carried out by Butnaru et al. [25] on the gas barrier properties of nanoclay also agree with the results that it has the ability to increase the gas barrier properties of the polymer. Here, the studies were performed on chitosan film incorporated with rosehip seed oil and Cloisite 30B nanoclay. The OP of the control chitosan film was 67 mL/m^2^.per day and this value increased up to 134 mL/m^2^.per day with the incorporation of Cloisite 30B nanoclay. In comparison to the chitosan film, the inclusion of Cloisite 30B nanoclay improves CO_2_ barrier characteristics, resulting in 50% lower CO_2_ permeability values [25]. Thus, nanoclays overall decrease the permeability of moisture and CO_2_ by 30% and 50%, respectively. The shelf life of meat products is increased and lipid oxidation is decreased due to the presence of nanoclay [33]. Thus, the structure of the nanoclay embedding in the biopolymer matrix increased the gas barrier and water vapour barrier properties of the packaging material.

### Antimicrobial properties of nanoclays-containing bio-based packaging materials

**Fig. 5:**
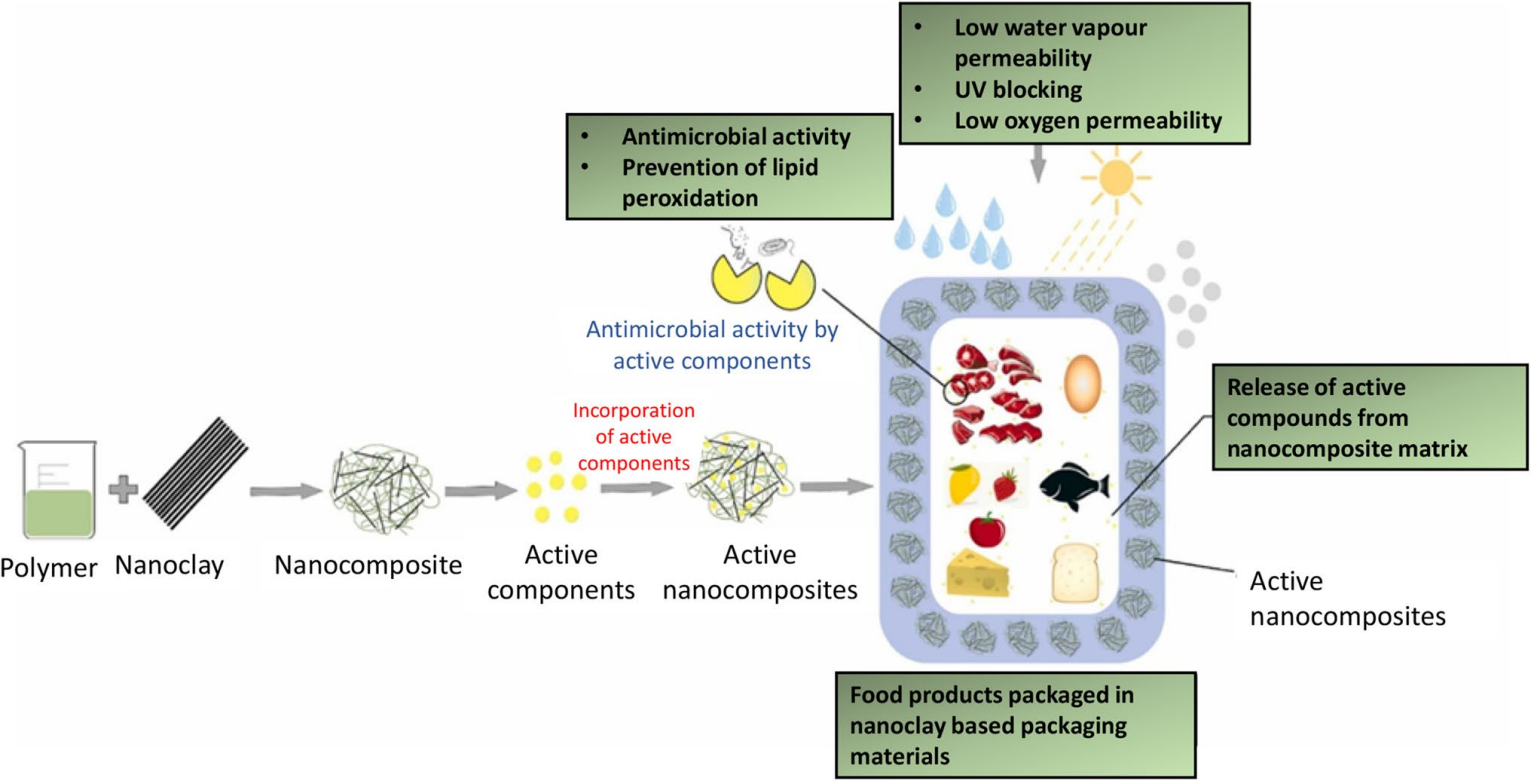
Schematic representation of developing active nanocomposites using nanoclay and antibacterial agents [71]

Antimicrobial packaging is fast evolving as a result of rising public awareness and desire for long-lasting active packaging that can preserve the quality and shelf life of foods and products. The incorporation of extremely effective antibacterial agents has been a crucial packaging advancement trend. Antimicrobial chemicals must be impregnated into the packing film to prevent or eradicate pathogenic bacteria that cause food disease and deterioration [26]. Some food packaging materials containing nanoclay have antimicrobial activity mainly depending upon the inserted antimicrobial agents such as essential oils, i.e. thyme [70] and *Carum copticum* essential oils [61] as further discussed in Table 4. The fabrication of nanoclay-based active food packaging films to improve antimicrobial activity can be seen in Fig. 5. However, some of the nanoclays Cloisite C30B [25] and organically modified nanoclays also have an antimicrobial activity that has great potential in food packaging applications.

**Table 4:** Effect of the nanoclay on the controlled release of the antimicrobial agents from the bio-based packaging films

Serial no	Type of nanoclay	Packaging materials	Concentration of nanoclay	Antimicrobial activity	References
1	MMT	Chitosan and nanoclay	1, 3, and 5 wt%	Best antimicrobial activity was observed in chitosan 3 wt%–nanoclay 1 wt% film to prevent microbial growth on Gouda cheese	[48]
2	Nanoclay Cloisite® Na +	Pectin– *Carum copticum* essential oils–β-carotene	0.05 W/V%	The inhibition zone for pectin–nanoclay film was 5.4 ± 0.43 mm (*B. cereus*) and 5.13 ± 0.3 mm (*E. coli*) Inhibition zone for Pectin–nanoclay– *Carum copticum* essential oils–β-carotene was 34.83 ± 3.11 mm (B. cereus) and 20.6 ± 0.81 mm ( *E. coli*)	[61]
3	MMT Cloisite 30B	Chitosan-rosehip seed oil	3 wt%	Chitosan film showed antimicrobial activity reduction of 65% for *S. typhimurium*, 73% for *E. coli*, and 82% for *B. cereus* Antimicrobial activity was increased for *S. typhimurium* and *B. cereus* when rosehip seed oil was added Bacterial inhibition was observed in all three bacteria in the packaging film containing Cloisite C30B	[25]
4	MMT	Poly(lactic acid)—Thymol	2.5 and 5 wt%	8 wt.% thymol and 2.5 wt.% MMT increased the antibacterial activity against *E. coli* (49.3 ± 0.5% cell viability) and *S. aureus* (51.6 ± 0.5% cell viability), where the control films had a 100% cell viability for both bacteria	[55]
5	MMT	Sweet potato–thyme essential oil	3%w/w	There was no antimicrobial activity observed in the film containing starch and against *E. coli* and *S. typhimurium* Increased antimicrobial activity (zone of inhibition) against *E. coli* (10 mm) and *S. typhimurium* (7 mm) on the 6% v/v thyme essential oil– MMT and starch	[70]

*MMT* montmorillonite

**Fig. 6:**
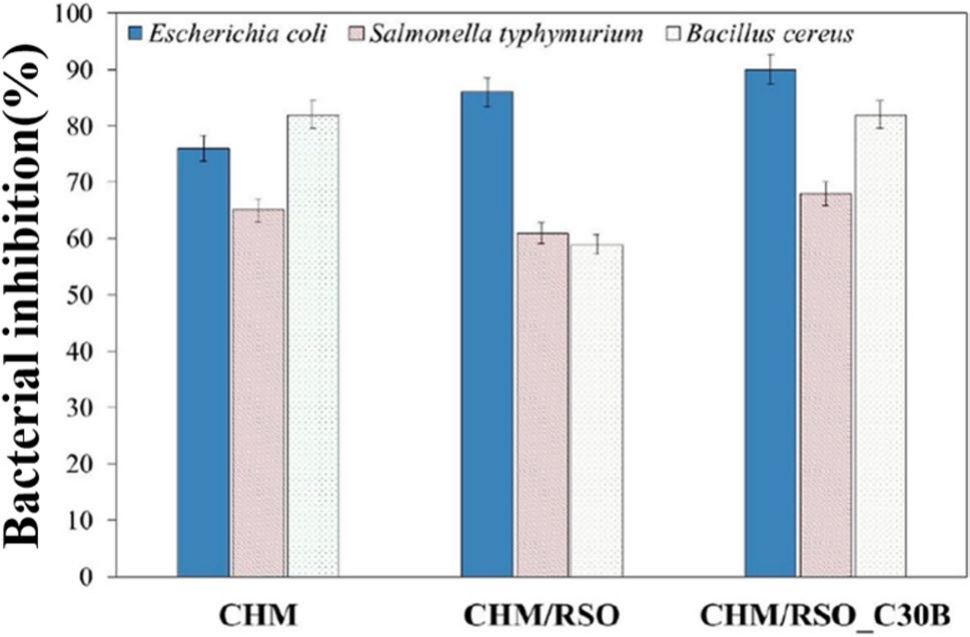
The antibacterial activity (% of bacterial inhibition) of Cloisite C30B incorporated bionanocomposites against *E. coli*, *S. typhimurium,* and *B. cereus* [25]

Butnaru et al. [25] created a packaging film based on chitosan-rosehip seed oil with the incorporation of nanoclay MMT Cloisite C30B (C30B) by the emulsion technique for food preservation application. Three different films were formulated as chitosan (CHM), chitosan–rosehip seed oil (CHM/RSO), and chitosan–rosehip seed oil–3 wt% Cloisite C30B (CHM/RSO/C30B). The antimicrobial activity of these three films against *E. coli*, *S. typhimurium,* and *B. cereus* are presented in Fig. 6. As shown in this figure the pure chitosan film showed antimicrobial activity reduction of 65% for *S. typhimurium*, 73% for *E. coli*, and 82% for *B. cereus*. When rosehip seed oil was introduced, the antibacterial inhibition for *S. typhimurium* and *B. cereus* was decreased. However, bacterial inhibition was observed in all three bacteria in the packaging film containing Cloisite C30B. Thus, it indicates the antimicrobial activity of Cloisite C30B [25].

Bioactive antimicrobial agents have been added with the combination of nanoclay for antimicrobial food packaging materials. Ramos et al. [55] incorporated MMT and thymol into a poly(lactic acid) matrix. In this study, the films were made of 8 wt.% thymol and different concentration of MMT (2.5 and 5 wt.%) using the melt blending method. The antimicrobial activity was determined in *E. coli* and *S. aureus* at 4 °C, 24 °C, and 37 °C for 3 h and 24 h using the direct contact method, and the bacterial cell survival was expressed as the percentage of colony-forming unit (CFU). The highest antimicrobial activity was observed in 8 wt.% thymol and 2.5 wt.% MMT; *E. coli* (49.3 ± 0.5% cell viability 37 °C, 24 h) and *S. aureus* (51.6 ± 0.5% cell viability 37 °C, 24 h), were the control films had a 100% cell viability for both bacteria. Additionally, the presence of nanostructured networks created in binary and ternary systems enables the regulated release of thymol into the food simulant [55].

PLA–2.5 wt.% MMT (*E. coli*: 79.4 ± 1.2% cell viability 37 °C, 24 h and *S. aureus*: 84.6 ± 0.9% cell viability 37 °C, 24 h) and PLA–5 wt.% MMT (*E. coli*: 71.7 ± 1.2% cell viability 37 °C, 24 h, and *S. aureus*: 89.8 ± 2.0% cell viability 37 °C, 24 h) showed similar antimicrobial activity in most of the tested conditioned. As shown by both of the above studies, organically modified nanoclay has shown antimicrobial activity against both Gram-positive and Gram-negative bacteria by rupturing the cell membranes themselves. This effect is attributed to the quaternary ammonium groups in nanoclays, which are capable of reacting with the lipids and proteins in microbial cell walls [55].

**Fig. 7:**
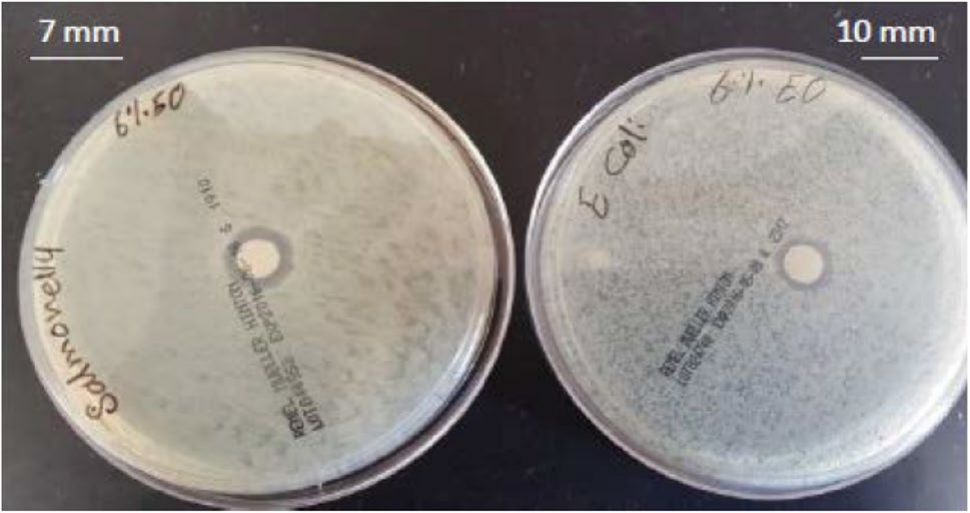
Antimicrobial activity through inhibition zones of starch–MMT–6% v/v thyme essential oil bionanocomposite against *E. coli* and *S. typhimurium* [70]

Antimicrobial studies were also conducted on MMT nanoclays where it was combined in a sweet potato starch matrix with the natural antimicrobial agent thyme essential oil. There was no antimicrobial activity observed in the film containing starch and MMT (3% w/w) against *E. coli* and *S. typhimurium.* However, there was increased antimicrobial activity (zone of inhibition) against *E. coli* (10 mm) and *S. typhimurium* (7 mm) on the 6% v/v thyme essential oil- MMT and starch film as shown in Fig. 7. These results indicate that there is no antimicrobial activity in MMT; however, it allows the release of the antimicrobial agent into the matrix, thus resulting in high antimicrobial activity. In addition, the addition of thyme essential oil to the film reduced the population of *E. coli* and *S. typhi* on fresh baby spinach leaves to below detectable levels within five days, but the control samples lacking essential oil maintained roughly 4.5 Log CFU/g [70]. Although it can be observed that nanoclay does not have antimicrobial activity unless it is modified, it does not block the release of the antimicrobial agents into the surroundings. Thus, the antimicrobial activity in not prevented through the addition of nanoclay. Nanoclay can be modified as organonanoclay as discussed previously, this results in the antimicrobial activity for the nanoclay. Further, controlled release of the antimicrobial agents maybe also observed which prolong the antimicrobial activity of the packaging material.

### Effect of nanoclays on the biodegradation of nanoclays-containing bio-based packaging materials

Biodegradation is an important quality of the packaging material. This is of considerable interest because of its various unique capacities to be degraded by the environment, to be created from natural resources, or be compostable to enable their removal from the environment once their working life has ended [72]. Multiple research have been conducted to investigate the influence of nanoclay on the biodegradable process.

Biodegradation was assessed for 4 and 18 days by a soil burial test for a food packaging material developed from pullulan, tempo cellulose nanofibrils, and MMT. Results from this study showed that the incorporation of MMT reduces the rate of biodegradation of the packaging film. This is due to the fact that there is a formation of strong hydrogen bond interactions between the pullulan matrix's hydroxyl groups and MMT nanoclay that increase the surface area. This resulted in the increment of the matrix’s cohesiveness and decreases water sensitivity [65].

PLA a biodegradable and compostable polymer was used to evaluate the biodegradable nature of nanoclay. The influence of organo-modified MMT Halloysite nanotubes and Laponite^®^ RD on the biodegradation of PLA was examined with an in-house designed direct measurement respirometer system and an analysis of evolved CO_2_ method. The results of the biodegradation experiments demonstrate that the biodegradation phase of nanoclay-containing films began earlier than that of pure PLA films [73].

**Fig. 8:**
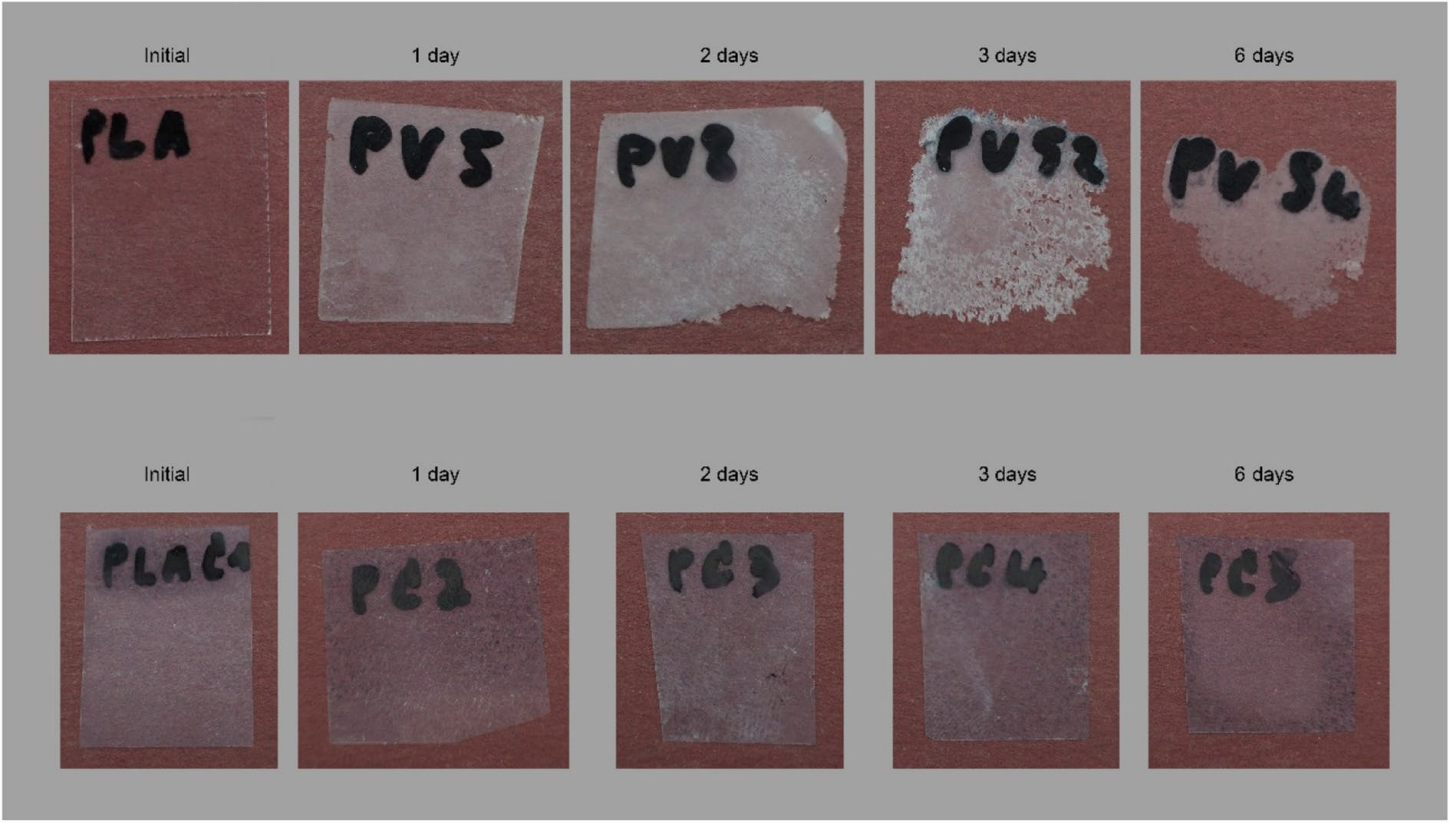
Proteinase K-based chemical biodegradation of PLA film (UP) and PLA + 4% nanoclay film (down) during 6 days

The same results were observed in a study performed by Oliver-Ortega et al. [40] where the biodegradation of PLA with BT nanoclay films was observed by using proteinase K as a biodegradation agent. It was observed that the inclusion of nanoclays resulted in a decrease in biodegradable capacity as shown in Fig. 8. Nanoclay must not hinder the biodegradation of bio-based food packaging materials, as this is one of the most crucial qualities when producing a novel food packaging material. However, limited studies are seen in the biodegradation studies on bio-based packaging material and nanoclay.

### Safety of nanoclays on nanoclays-containing bio-based packaging materials: migration of nanoclays

It is possible for nanoparticles to interact with food components during processing, storage, and distribution, resulting in nanoparticle migration into food products. Migration could also occur if the packaging is not disposed of properly and is left in the environment exposing plants and wildlife to nanoparticles from composite materials. It has also been noted that even if food packaging containing nanoclay is recycled, physical alteration such as shredding and drilling could also result in migration from the packaging to the environment affecting human life and wildlife [74]. The possibility of nanoparticle migration from the packaging material made of nanocomposite into the food is quite unlikely as long as the nanoparticles are thoroughly lodged within the polymer matrix. However, nanoparticles may come into touch with food due to mechanical impact. As a result, it is crucial to investigate nanoparticle migration in the packaging material. Nanomaterials migrate through Fick’s diffusion, similar to how conventional polymer additives operate, or through alternative mechanisms based on material stress, namely breakdown of the polymer matrix through mechanical abrasion, material fatigue, UV exposure, hydrolysis, or swelling interactions. Purely chemical release can also happen when nanoparticles dissolve into ionic species in a polymer and then release those ions into food. Due to weak bonding forces and mechanical stress, nanolayers on top of polymer surfaces may desorb nanoparticulate fragments [75]. The chemical properties such as the vapor pressure, polarity, molecular size, and structure influence the rate of migration and the migration of nanoparticles. Further, the concentration of the nanomaterial presence also influences the migration of the nanomaterials [76].

Tests have been conducted to determine if the recycling of food packaging containing nanoclays poses a threat to the environment. It was found that during shredding, a lower concentration of airborne particles was reported when a polypropylene nanocomposite plate was shredded in comparison to the air quality during the shredding of neat polypropylene (PP). This is due to the fact that the particles are still anchored within the matrix polymer, which implies that recycling nanocomposite materials should not result in higher health concerns for the environment (and those living there) than recycling regular PP. Along with mechanically induced release processes, nanoparticles migrate by desorption, diffusion, and dissolution. Desorption occurs when there is a weak bonding between the matrix and nanoparticles. A concentration gradient also has the potential to induce migration of nanoparticles from a nanocomposite by diffusion, whilst dissolution occurs when internally embedded and/or surface-bound nanoparticles produce ions that migrate into the product. As non-mechanical migration can occur due to many different variables, it is necessary to take an individual approach when testing their safety. This would include the structure of the nanocomposite itself, the conditions it must withstand, and the properties of the product that will be held in it [77].

As it is apparent that migration does occur in nanocomposite food packaging, experiments have been conducted to assess the toxicity risks associated with this migration. When studies of migration is performed the migration of different chemicals of nanoclay is determined. For instance, nanoclay is composed of phyllosilicates which contain groups of minerals such as talc (Mg_3_[Si_4_O_10_(OH)_2_]), mica (KAl_2_[AlSi_3_O_10_(OH)_2_]), kaolin (Al_2_[Si_2_O_5_(OH)_4_]), MMT (Mg_0.33_Al_1.67_[Si_4_O_10_(OH)_2_](Ca, Na)x(H_2_O)_n_), serpentine (Mg_3_[Si_2_O_5_(OH)_4_]) or sepiolite (Mg_4_[Si_6_O_15_](OH)_2_ 4H_2_O) [78]. When performing migration studies on nanoclay, the presence of the different metals in the food stimulants is determined through inductively coupled plasma mass spectrometry (ICP-MS). Echegoyen et al. (2016) studied the migration of nanoclays from two commercialized low-density polyethylene nanocomposite bags. Migration was studied through the use of two food stimulants, (ethanol 10% and acetic acid 3%) at two test conditions 40 ºC for 10 days and 70 ºC for 2 h. To mimic the reuse of the bags by consumers, the tests were carried out three times in each condition on both the ‘Debbie Meyer’ bags and the ‘Aisaika’ bags [79]. The migration solutions collected at the end of these tests were analysed by ICP-MS and the aluminium (aluminium is one of the main elements in nanoclay), which migrated into the solutions as a part of nanoparticles and in dissolved form was equated. A maximum migration value was observed in the ‘Aisaika’ bags at 51.56 ng/cm^2^ and 24.12 ng/ cm^2^ for the ‘Debbie Meyer’ bags, the migration levels appeared higher than the latter sample due to their higher load of nanoclay in the composite, with this in consideration, the relative migration values were similar for both bags. It was also found that the condition which induced the highest levels of migration in both samples was acetic acid 3% for 10 days at 40 ºC. Overall, scanning electron microscopy coupled to energy-dispersive X-ray diffraction (SEM–EDX) analysis confirmed the presence of nanoparticles in the solutions, although the levels of migrated nanoclay were ‘very low’ compared to the established legislative migration limit of 10 mg/dm^2^ as mentioned in detail in section 8.

Further, studies on food stimulants to assess the migration of nanoclays were carried out by Connolly et al. (2019). The nanocomposite packaging materials were designed using poly(lactic acid) and organically modified clays (organoclays). The migration studies were performed in the food simulants vegetable oil, and aqueous media which were assessed for 10 days at an incubation temperature of 40 °C. These studies indicated that the maximum migration was 0.88 ± 0.44 mg/dm^2^ for the nanocomposite films, whereas the excepted level of migration is 10 mg/dm^2^ [80]. Further, toxicity studies were also performed on the human skin cells (HaCaT immortalized human keratinocytes), where significant cell viability was not observed. These results indicate that the migration level of the organoclay is very low, while it is not toxic to the human skin. There are very limited studies of migration and toxicity on bio-based polymers (Table 5); thus, studies on plastic have been discussed. Further, studies should be performed in migration and in vitro cell culture studies before nanoclay is to be industrialized, used by human population, and released into the environment.

**Table 5:** Studies on the migration of the nanoclay from bio-based packaging material

Serial no	Type of nanoclay	Packaging materials	Stimulant/food product	Test temperature and time	Migration levels	References
1	Nanoclay	LDPE ‘Debbie Meyer’ Packaging bag	10% ethanol	40 °C for 10 days	7.53 ± 1.62 ng/cm^2^	[79]
			10% ethanol	70 °C for 2 h	2.10 ± 0.85 ng/cm^2^	
			3% acetic acid	40 °C for 10 days	24.14 ± 2.90 ng/cm^2^	
			3% acetic acid	70 °C for 2 h	7.97 ± 0.84 ng/cm^2^	
2	Nanoclay	LDPE ‘Aisaika’ Packaging bag	10% ethanol	40 °C for 10 days	14.42 ± 1.66 ng/cm^2^	[79]
			10% ethanol	70 °C for 2 h	2.71 ± 0.93 ng/cm^2^	
			3% acetic acid	40 °C for 10 days	51.65 ± 2.90 ng/cm^2^	
			3% acetic acid	70 °C for 2 h	8.09 ± 3.15 ng/cm^2^	
3	Nanoclay	Poly(lactic acid)	Vegetable oil, Aqueous media	40 °C for 10 days	0.88 ± 0.44 mg/dm^2^	[80]
4	MMT non-polar-modified organoclay	Polypropylene	Isooctane	20 °C for 48 h	17.86 ± 0.14 mg/dm^2^	[81]
			10% ethanol solution	20 °C for 48 h	0.42 ± 0.38 mg/dm^2^	
			3% acetic acid	40 °C for 10 days	0.46 ± 0.16 mg/dm^2^	
5	MMT organic modifier containing polar groups	Polypropylene	Isooctane	20 °C for 48 h	16.62 ± 0.11 mg/dm^2^	[81]
			10% ethanol solution	20 °C for 48 h	0.66 ± 0.32 mg/dm^2^	
			3% acetic acid	40 °C for 10 days	0.56 ± 0.04 mg/dm^2^	
6	Laponite	LDPE	Novachem surfactant solution	60 °C for 10 days	0 mg/dm^2^	[82]
7	BT	Poly(lactic acid)	Water	20 °C for 10 days	< 1 mg/dm^2^	[50]
			Acetic acid 3% w/v	20 °C for 10 days	< 1 mg/dm^2^	

*BT* bentonite, *MMT* montmorillonite; *LDPE* low-density polyethylene

## Legislation related to nanoclays-containing packaging materials

Nanomaterials are also considered as materials to produce novel food products and so are covered by the novel food regulation (Regulation (EU) 2015/2283) [83]. It is stated the engineered nanomaterials presented in food must be outlined in the list of ingredients and nanomaterials used in food contact materials must be explicitly authorized and a specific risk assessment of the nanomaterial must be carried out [84].

More legislation that affects the use of nanoclay in food packaging is the Corrigendum to Commission Directive 90/128/EEC of 23 February 1990 [83] relating to plastics materials and articles intended to come into contact with foodstuffs. This specifies that the total migration limit of material constituents to the food product for plastic-based food packaging (such as plastic-derived nanocomposites) is 10 mg d/m^2^ of the packaging material's surface area, although in 500 mL to 10 L food containers where it is impossible to measure the surface area in contact with the products, the limit is extended to 60 mg per kg of foodstuffs. Studies have since been conducted on the migration of certain nanoclay constituents from nanocomposites to food products and it was found that BT, kaolin, and hexadecyltrimethylammonium bromide-modified MMT are recognized as safe by the FDA as a food contact substance [74].

The European Commission announced the European Union novel foods regulation EU No. 2015/2283 [85], this legislation addressed specific information requirements and the authorisation for specific uses for nanomaterials and their safety assessments. In this, the European Commission outlines that nanomaterials are comparative to normal ingredients which might be toxic in some cases and that a risk assessment must be carried out and followed in each case. The European Food Safety Authority has designed and published guidance on risk assessment of the application of nanoscience and nanotechnology in the food and feed industry. It also outlines the physicochemical properties, exposure assessment, and hazard characterization of nanomaterials [86].

The migration restrictions of specific chemical substances, or a set of chemicals, were explained in Commission Regulation (EU) No 10/2011 [87]. The chemical has a detection limit of 0.01 mg/ kg of food or food simulant. Commission Regulation (EU) 2016/1416 [87] and Commission Regulation (EU) 2020/1245 [88] revised this regulation at various stages. According to authorities, unplasticized polymers have a specific migration level of 0.05 mg/kg [87]. When utilized as an additive in plastic food contact materials, MMT clay treated with hexadecyltrimethylammonium bromide does not pose any safety issues, according to the authorities, and no migration is detected when the nanoparticle range is less than 100 nm [88].

## Conclusion and future trends

The utilization of bionanocomposites-based food novel packaging material has become of great interest in the food packaging industry, mainly due to its importance in the increment of biopolymer properties such as mechanical properties and the barrier properties of the packaging material. Despite the fact that most nanoclays lack antibacterial activity, they can regulate the release of antimicrobial chemicals integrated into the packaging material. In addition, nanoclays reduce the rate of biodegradation of the packaging materials. Nevertheless, most importantly, when considering the limited studies performed the migration levels of nanoclay are very low while it is non-toxic. Regardless, given the rapid growth of nanomaterial-based food packaging on an industrial scale, it is critical to emphasize the importance of conducting short and long-term toxicity studies in both the environment and humans in order to ensure consumer safety. Thus, further studies on biodegradation, migration, and toxicity of packaging material with bio-based polymers and nanoclay are recommended. With the great importance of nanoclay in enhancing the properties of polymers its industrial applications are very limited. Nanoclay is the first nanomaterial which was industrialized decades ago and it is now critical to promote bio-based nanoclay packaging materials to the food industry. In future, nanoclay will have potency of develop nontoxic, low-cost, environmental friendly food packaging materials with enhanced properties.

## Data Availability

Data sharing not applicable–no new data generated.
